# Clinical response to azacitidine therapy depends on microRNA-29c (miR-29c) expression in older acute myeloid leukemia (AML) patients

**DOI:** 10.18632/oncotarget.7172

**Published:** 2016-02-03

**Authors:** Aleksandra Butrym, Justyna Rybka, Dagmara Baczyńska, Rafał Poręba, Kazimierz Kuliczkowski, Grzegorz Mazur

**Affiliations:** ^1^ Department of Hematology, Blood Neoplasms and Bone Marrow Transplantation, Wroclaw Medical University, Wroclaw, Poland; ^2^ Department of Physiology, Wroclaw Medical University, Wroclaw, Poland; ^3^ Department of Forensic Medicine, Molecular Techniques Unit, Wroclaw Medical University, Wroclaw, Poland; ^4^ Department of Internal, Occupational Diseases, Hypertension and Clinical Oncology, Wroclaw Medical University, Wroclaw, Poland

**Keywords:** miR-29c, acute myeloid leukemia, azacitidine, response to therapy, expression

## Abstract

Acute myeloid leukemia (AML) is a heterogeneous disease with different clinical course and prognosis. microRNA-29 (miR-29) family of non-coding small RNAs can play an important role in pathogenesis of AML, but also can influence response to therapy.

The purpose of the study was to evaluate miR-29c expression in AML patients in relationship to clinical parameters and response to chemotherapy, including azacitidine.

**Materials and Methods:**

miR-29c expression has been analyzed using RT-PCR in 95 bone marrow specimens from newly diagnosed AML patients in comparison to 20 healthy subject.

**Results:**

We showed up-regulated miR-29c expression in AML patients which was linked also to higher risk of disease relapse after achieving complete remission. In subset of elderly AML patients treated with azacitidine, low miR-29c expression at diagnosis correlated with good response to therapy.

**Conclusions:**

miR-29c is of prognostic value and influences response to azacitidine treatment in older AML patients.

## INTRODUCTION

Acute myeloid leukemia (AML) is a hematopoietic disorder characterized by increased, uncontrolled proliferation of progenitor cells and their blocked differentiation. In AML there is a bone marrow blast count over 20% and poor prognosis of patients with this diagnosis. In recent times there has been a huge improvement in the therapy of adult AML, but still it is difficult to find optimal therapeutic option. The population of AML patients is very heterogenous and at the moment of disease diagnosis all patients are stratified to different risk groups with different prognosis. Although there are many prognostic factors, particularly molecular and cytogenetic, we need the new one for better patient allocation and individual treatment. The issue of AML treatment and chemotherapy dosing specially regards older patients, in which we do not have standard treatment, thus overall survival and response rates remain poor. Recently the results of international phase III trial of azacitidine in the therapy of older AML patients has been published, showing azacitidine as a good option for this group of patients [1].

MicroRNAs (miRs) are short non-coding RNAs, 19–25 nucleotides long, which have an important role in regulation of fundamental processes of cell development, differentiation, growth and proliferation. Dysregulation of miRs expression can contribute to pathogenesis and prognosis of many cancers, including AML [2, 3].

MiR-29c belongs to miR-29 family, together with miR-29a and miR-29b. All mature miR-29 have highly homologous sequences, but different isoforms of miR-29 family can have different functions. In AML miR-29 has been shown to act as tumor suppressors and regulators of cell proliferation and apoptosis, thus it has an important role in normal and malignant haematopoiesis [4]. Results of miR-29 expression in AML patients has been so far quite different and linked to heterogeneous subtypes of AML [5], but no reports are known regarding the role of miR-29 in response to new kind on AML treatment with azacitidine.

The purpose of this study was to evaluate miR-29 expression in newly diagnosed AML patients in comparison to healthy controls, relationship with clinical features of the disease and analysis of any potential influence of miR-29 expression on efficacy of azacitidine in subsets of older AML patients.

## RESULTS

**Figure 1:**
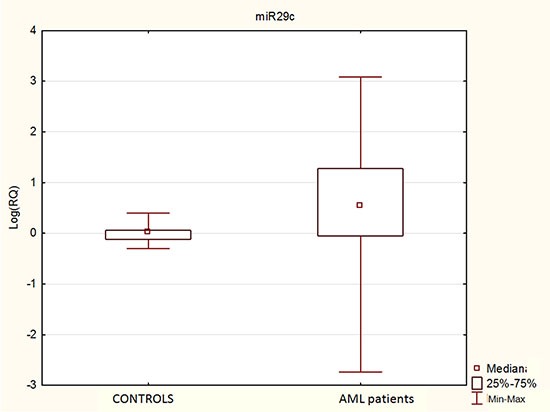
miR-29c expression in AML patients comparing to control group

We showed higher miR-29c expression in AML patients in comparison to control healthy group (*p* < 0.0001), Figure 1.

Experiments performed after induction chemotherapy in AML patients showed significant change in miR-29c expression (*p* < 0.05). Achieving complete remission (CR) after initial treatment was linked to drop in miR-29c expression.

Patients who achieved CR with higher than median miR-29c expression at diagnosis and were at higher risk of relapse (*p* = 0.02).

**Figure 2:**
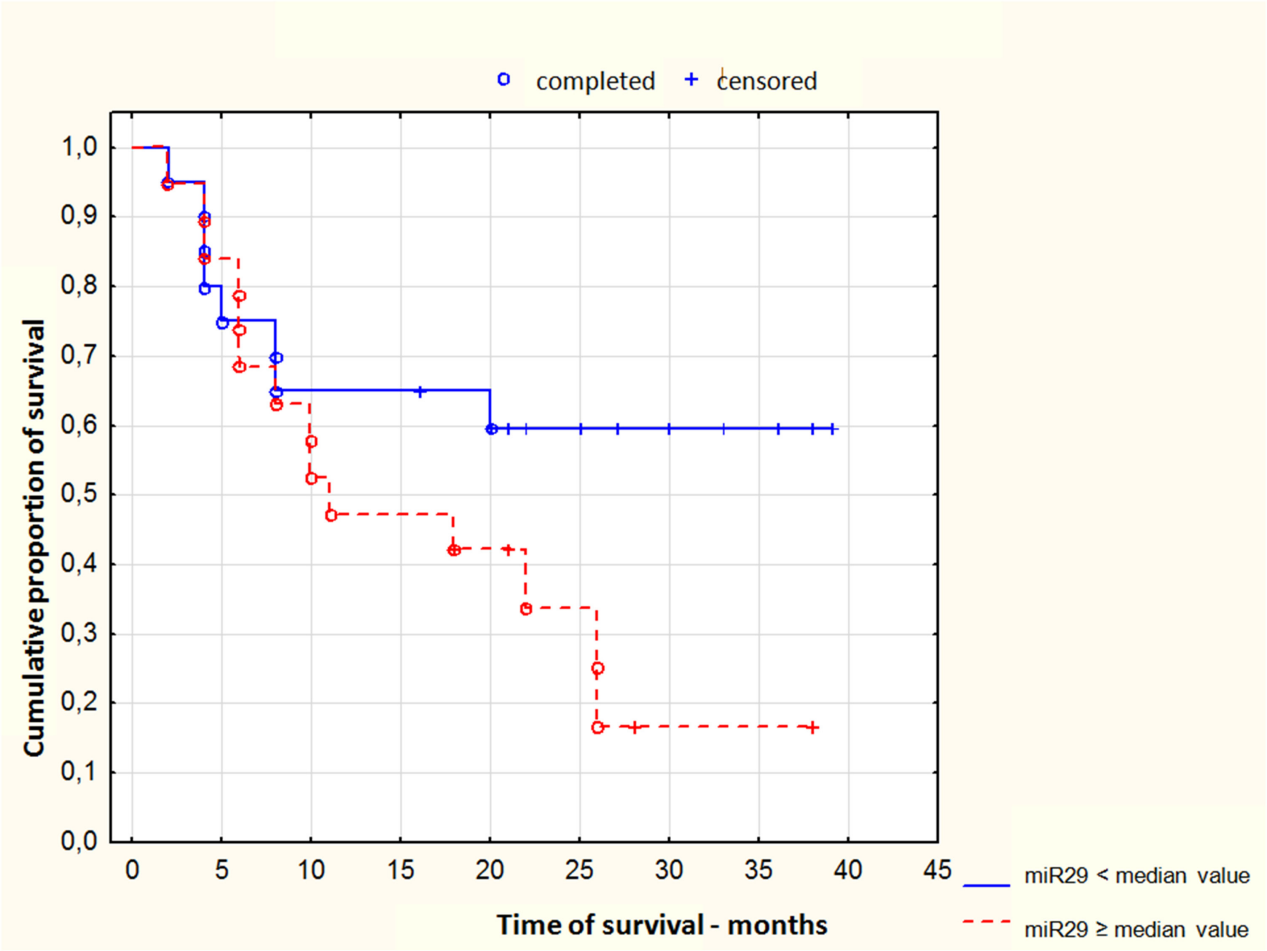
Kaplan-Meier curve of probability of AML patients survival depending on miR-29c expression at the moment at diagnosis Me - median value of expression.

miR-29c expression at diagnosis also determined patients survival (*p* = 0,03951). Lower miR-29 expression was associated with prolonged survival in comparison to higher expression in AML group. Figure 2.

### Patients treated with azacitidine

**Figure 3:**
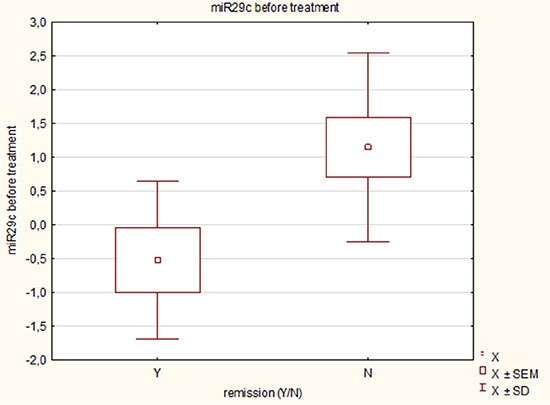
Response to azacitidine treatment depending on miR-29c expression at diagnosis in acute myeloid leukemia patients

We observed, that lower miR-29c expression at diagnosis in AML patients treated with azacitidine was correlated with good response to azacitidine and remission achieving (*p* = 0.03). Figure 3.

**Figure 4:**
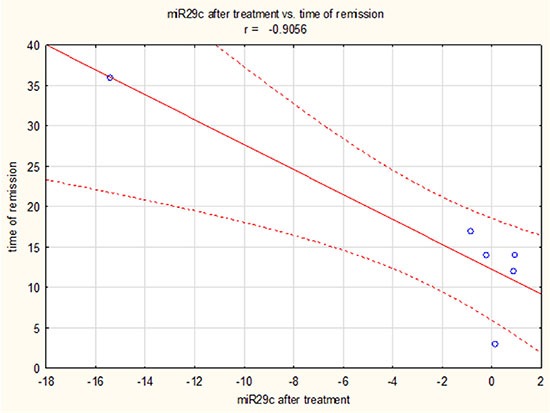
Duration of complete remission in AML patients depending on miR-29c expression after chemotherapy

The same, time of remission duration was longer in patients who had low miR-29c expression after chemotherapy (*p* < 0.05, coeff .905581) Figure 4.

## DISCUSSION

AML is heterogenous disease with different clinical course and prognosis. In this disease it is very important to determine risk factors for this population of patients, for good stratification and treatment. The group of older patients in AML has particularly poor prognosis and still remains without optimal and effective therapy due to possible toxicity and comorbidities. In the last few years low dose therapy with hypometylating agents, such as azacitidine and decitabine has shown some promising results, prolonging overall survival with better quality of life [1, 6, 7]. As to our knowledge, our study is a first report linking miR-29c expression to the results of azacitidine therapy in AML patients.

It has been already proved, that miR-29 family had been involved in the regulation of many important processes leading to cancer genesis and progression, acting mainly as tumor suppressor [8]. In this study we showed significantly higher miR-29c expression in AML patients comparing to healthy controls and high expression was linked to increased risk of disease relapse. Deregulation of miR-29 family expression can be also involved in AML pathogenesis but it differs depending on scientific reports. Wang et al. reported decreased miR-29a in adult AML patients and underlined its role in monocytic and granulocytic differentiation [9]. Contrary, Han et al. demonstrated high expression levels of miR-29a in similar patient population, with its impact on self-renewal capacity in hematopoietic progenitors [10]. Up-regulated expression of miR-29c in AML with aberrant cytoplasmic NPM1 localization was found by Garzon et al. [11].

As miR-29 targets DNA methyltransferases (DNMTs) and thus has its epigenetic effect on AML cells, we tended to verify relationship between therapy with hypomethylating agent such azacitidine and miR-29c expression in subset of older AML patients [7, 12]. Our results indeed confirmed that low expression of miR-29c in elderly AML patients was linked to good response to therapy and prolonged duration of complete remission. Therapy with azacitidine usually requires 3 to 6 cycles to see any effectiveness of the drug and there are patients who will not respond to azacitidine. Determination of miR-29c expression at diagnosis could help to avoid unnecessary costs and ineffective therapy. In 2010 Blum et al. showed that clinical response to another hypomethylating agent decitabine in older AML patients was correlated with miR-29b expression [7]. The miR-29 family has complementary structure to 3′-UTR of methyltransferases DNMT3a and DNMT3b [4]. In cases with low miR-29b expression, restoration of miR-29b in AML cells reduced DNA hypermethylation and induced re-expression of previously hypermethylated suppressor genes by direct binding to DNMTs [12]. Blum et al. continued their investigation on miR-29 family and bortezomib and decitabine treatment in AML [13]. They showed possibility of up-regulation of miR-29b expression by bortezomib and increased sensitivity of tumor cells to decitabine.

Concluding, we are the first to show, that miR-29c in older AML patients can determine response to new kind of antileukemic therapy with azacitidine. As this treatment is well tolerated and safe in this patients population, it is important to have a tool for selection of patients who will benefit from therapy. miR-29c can have predictive value for treatment response and survival in acute myeloid leukemia patients, but it should be validated as the stratification tool in bigger AML cohort.

## MATERIALS AND METHODS

### Patients characteristics

The study included 95 patients (aged 60.2 ± 15.0, 22–90, Male = 61%) with newly diagnosed AML. Samples of the bone marrow for miR-331 expression analysis were collected before start of chemotherapy and repeated after completed induction chemotherapy (40 patients). Patients were treated in the Department of Hematology, Blood Neoplasms and Bone Marrow Transplantation of Wroclaw Medical University, Wroclaw, Poland. A control group of 20 healthy subjects was also taken into account (aged 64.2 ± 10.5, 39–80, Male = 65%). According to AML FAB classification, 7 patients had AML M0, 34 had M1, 29 had M2, 14 had M4 and 11 had M5. There were 73 patients with primary leukemia and 22 patients with leukemia secondary to myelodysplastic or myeloproliferative syndrome. Summary of patients’ characteristics is present in Table 1.

**Table 1:** Clinical characteristics of patients with AML

Characteristics	Cases
**Sex**	
Male	56
Female	39
**Age (years)**	
Range	22–90
Median	61
**FAB subtype**	
M0	7
M1/M2	63
M4/M5	25
**WBC (G/L)**	
Range	0.2–295
Median	14
**HGB g%**	
Range	5.8–13.1
Median	9.3
**PLT (G/L)**	
Range	2–310
Median	65
**Lactate dehydrogenase (LDH) U/l**	
Range	108–4565
Median	340
**Blasts in bone marrow**	
< 50%	35
≥ 50%	60
**Cytogenetics**	
Farorable	5
Intermediate	39
Unfavorable	51
**Chemotherapy**	
Intensive	56
Low dose	27
Best supportive care	12
**Molecular tests**	**Total 60 patients**
AML/ETO (positive/negative)	4/56
CBFb-MYH11 (positive/negative)	2/58
NPM1 (positive/negative)	7/53
FLT3/ITD (positive/negative)	13/47
**Complete remission**	
Yes (total)	51
Yes (after 1st line therapy)	36
No	44
**Duration of remission (months)**	
Range	2–54
Median	20
**Time to relapse (months)**	
Range	3–23
Median	12
**Survival (months)**	
Range	0–55
Median	3

After diagnosis, 56 patients were treated with standard induction intensive chemotherapy (daunorubicin plus cytarabine 3 + 7), 27 received low dose chemotherapy (low dose cytarabine or azacitidine) and 12 best supportive care only. After completion of induction therapy response to treatment was evaluated. CR was defined by Cheson criteria [14]. Also bone marrow samples were re-evaluated for miR-29 expression in 40 patients. Patients were followed up for median 21 month (range 1–40 months).

### AML patients treated with azacitidine

There were 17 newly diagnosed AML patients, not suitable for intensive chemotherapy, treated with azacitidine (4 women and 13 men, aged 65–90 years, median age 75 years). Azacitidine was administered 75 mg/m^2^ subcutaneously, on days 1–7 of 28-day cycles. Patients received 1–35 cycles of azacitidine (median 7 cycles). 6 patients achieved complete remission, 6 had partial response or disease stabilization and 5 did not respond to therapy. At the time of analysis, all patients, except one, were dead.

### Ethical statement

Research was carried out in compliance with the Helsinki Declaration. For the study approval of Bioethical Committee of Wroclaw Medical University was obtained. Written informed consent for study was obtained from all the participants.

### Isolation and expression analysis of microRNAs

Bone marrow mononuclear cells (PBMC) were isolated by Ficoll-Hypaque density gradient centrifugation. Total RNA and microRNA were extracted from collected AML mononuclear cells using mirVana^™^ miRNA Isolation Kit (Ambion) according to the protocol of the manufacturer. Then 5 μl total miRNA was used as a template into synthesis of cDNA using TaqMan MicroRNA Trasncription Reaction Kit (Applied Biosystems) and 3 μl specific miRNA primers from the TaqMan MicroRNA Assays (Applied Biosystems). Individual reaction was carried out in 15 μl total volume in thermal condition: 16°C for 30 min, 42° for 30 min, 85°C for 5 min. TaqMan MicroRNA Assays for miR-29 (hsa-miR-29), and RNU48 were used. The expression level of each microRNA was measured in relative real-time PCR method using TaqMan Gene Expression Assays and TaqMan Fast Universal PCR Master Mix (Applied Biosytems). All reactions were done in triplicate in a total volume of 20 μl on 96-well plates. The real-time PCR was performed on 7900HT Fast Real-Time PCR System (Applied Biosystems) under thermal cycling conditions: 20 s at 95°C and 40 cycles of 1 s at 95°C and 20 s at 60°C. For quantification, the samples were normalized against the expression of RNU48 miR. Relative quantification factors (RQ) for the examined miRs were calculated using ^ΔΔ^CT method.

### Statistical analysis

The differences in means of gene expressions between the study and the control patients were estimated using t-Student's test (for independent samples). To examine the time it takes for death and remission to occur, a Cox's regression was applied [15]. The difference between the gene expressions before and after treatment was estimated using robust regression and multivariate approach [16]. The computation was performed in R software [17] and based on the simulation technique known as Gibbs sampling in WinBUGS platform [18]. Kaplan-Meier survival curves were used to determine any significant relationship between miR-29c expression and clinical outcome. Results were considered statistically significant when *p* was < 0.05.
